# Sensitivity and specificity of recombinant proteins in *Toxocara* spp. for serodiagnosis in humans: Differences in adult and child populations

**DOI:** 10.1371/journal.pone.0208991

**Published:** 2018-12-13

**Authors:** Lucas Moreira dos Santos, Carolina Georg Magalhães, Paula de Lima Telmo, Michele Pepe Cerqueira, Rafael Amaral Donassolo, Fábio Pereira Leivas Leite, Guita Rubinsky Elefant, Luciana Farias da Costa Avila, Carlos James Scaini, Ângela Nunes Moreira, Fabricio Rochedo Conceição

**Affiliations:** 1 Department of Biotecnologia, Universidade Federal de Pelotas, RS, Brasil; 2 Department of Medicina Tropical, Universidade de São Paulo, São Paulo, SP, Brasil; 3 Faculty of Medicine, Universidade Federal do Rio Grande, Rio Grande, RS, Brasil; Instituto Butantan, BRAZIL

## Abstract

Toxocariasis is a neglected zoonosis that affects children and adults. Recombinant proteins have been widely investigated for diagnosis, achieving high sensitivity and specificity in an overall population; however, little is known about age as a factor in its application. This study aims to investigate the diagnostic potential of *Toxocara canis* TES-30 and TES-120 recombinant proteins in humans, differentiating between its performance in children and adults. Serum samples collected from children and adults seropositive to *Toxocara* spp. were tested with indirect ELISA using *T*. *canis* TES-30 and TES-120 recombinant proteins produced in *Escherichia coli*. While rTES-30 sensitivity was not affected by age (81.8% in children and 87% in adults), rTES-120 sensitivity severely decreased in children to only 63.6%, down from 95.7% in adults. Furthermore, the sensitivity of rTES-30 increased to 97.8% after Western blotting confirmation. High specificity (>94%) against other geohelminths was reported for both recombinant proteins. Our study favors the use of rTES-30 with total IgG as the primary antibody in an indirect ELISA assay as a tool for epidemiological human studies.

## Introduction

According to researchers at the Centers for Disease Control, toxocariasis is one of the most common neglected diseases in populations living in poverty [1]. This disease is transmitted via the infectious eggs of *Toxocara* sp. to definitive (dogs and cats) and paratenic (humans) hosts. In definitive hosts, the larvae mature to parasite adults and complete its life cycle: the host excrete non-infective eggs on the soil, which become infectious in the environment and is made available for infect new definite or paratenic hosts; however, in paratenic hosts, the larvae cannot reach adulthood, therefore, it migrates to tissue and organs and remain in the host until the immune system clear the infection or forces them into a state of arrested development [1]. For this reason, its symptomatology, in humans, varies according to larvae migration and its damage to the tissues; visceral larva migrans syndrome and covert toxocariasis are more common in children, while ocular toxocariasis and neurotoxocariasis can be found in both children and adults [2–5].

Clinical diagnosis is difficult as most manifestations (excluding ocular toxocariasis) lack characteristic symptoms, so the only viable diagnostic option is in the laboratory with Enzyme-linked immunosorbent assay (ELISA) and Western Blotting [6]. The production of proteins to be used in the laboratory assays requires in vitro larvae cultures, which demand excessive time, cost, and expertise to produce a viable product. Also, the *Toxocara* excretion-secretion protein family (TES) obtained from the larvae culture presents a number of cross-species reactions, requiring serum preabsorption that is troublesome in itself [7].

As an alternative, multiple studies have investigated the potential of recombinant proteins in the diagnosis of toxocariasis to reduce the time, cost, and cross-reactivity of native TES production [8–11]. Of the recombinant proteins previously studied, TES-30 and TES120 have been the most promising, achieving high sensitivity and specificity in the general population [8].

In children, however, these recombinant proteins have not been investigated thoroughly. Children are substantially more exposed to one of the most important risk factor for the disease: the geophagy habit. In addition, children are at risk for early infection and progression to ocular toxocariasis or/and neurotoxocariasis, potentially conferring permanent damage to vision and/or the brain, respectively, implicating toxocariasis as a major non-diagnosed health problem [4,12,13]. In addition, prior research has noted that the immune system of children responds differently than the immune system of adults to parasite infection, with lower expression levels of toll-like receptors and interleucin-5, both of which are important signaling pathways in the mature response against parasites [14–16]. This discrepancy in immune response could cause a deviation from the high sensitivity and specificity observed in adult studies.

Therefore, this study investigates the diagnosis potential of *T*. *canis* TES-30 and TES-120 recombinant proteins in children versus adults with the objective of validating an inexpensive diagnosis in the pediatric population. If effective, this diagnostic technique would represent an essential step for combating neglected aspects of the disease while permitting a fast and accurate diagnosis to reduce or prevent poor prognoses.

## Materials and methods

### Cloning and expression of recombinant proteins

**Fig 1:**
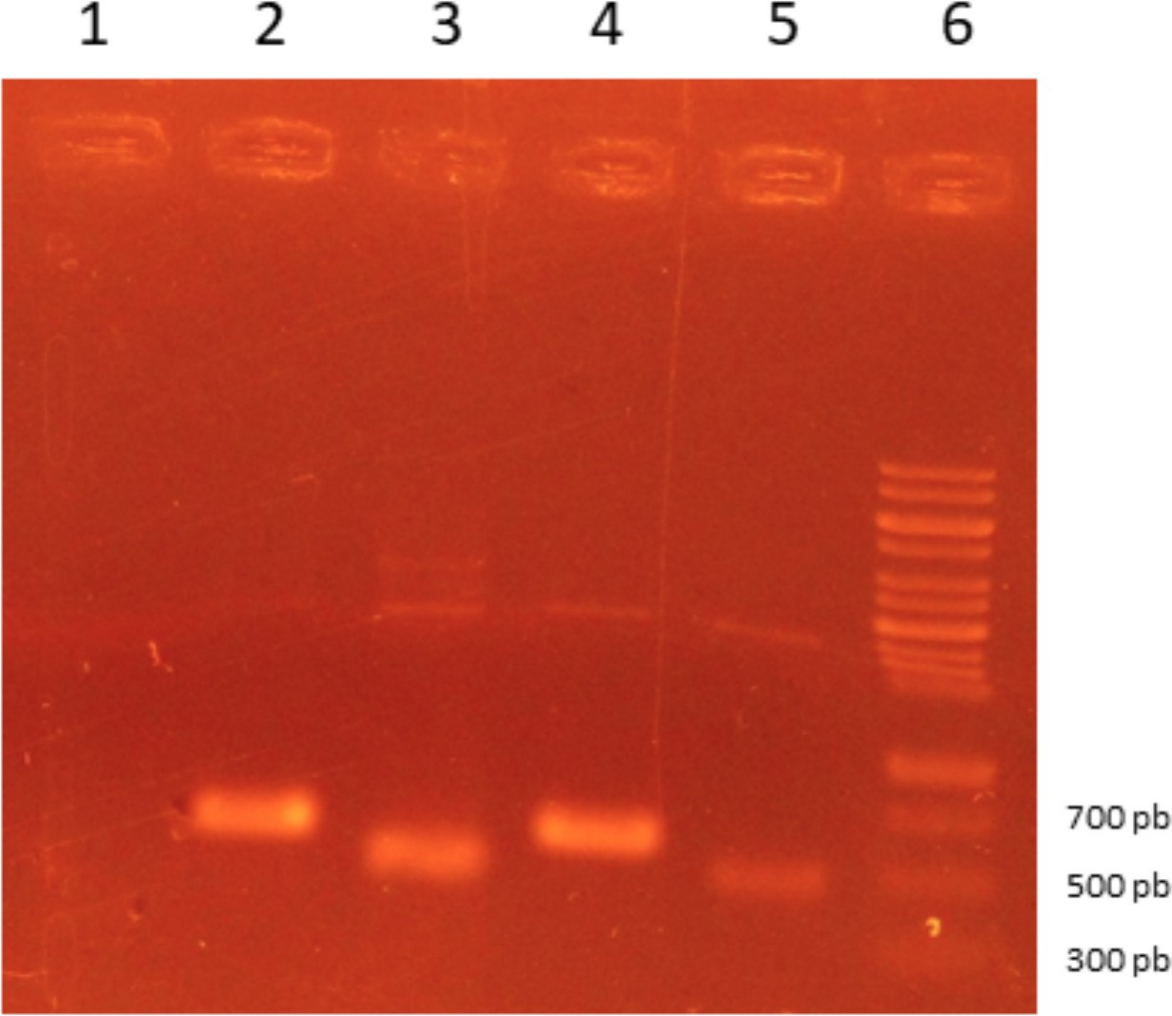
Agarose gel stained with ethidium bromide presenting PCR products. Lane 1: PCR Negative Control (PCR Mix). Lane 2: pAE rTES-30 Clone 1. Lane 3: pAE rTES-120 Clone 1. Lane 4: pAE rTES-30 Clone 2. Lanes 5: pAE rTES-120 Clone 2. Lanes 6: Ladder 100 pb (Ludwig Biotecnologia, Brazil).

Synthetic genes of TES-30 (Genbank access 4586556) and TES-120 (Genbank access 1103869) were cloned, using the restriction enzymes *Bam*HI e *Kpn*I, in the expression vector pAE [17] with a ligase reaction performed using T4 DNA ligase enzyme (Thermo Fisher Scientific, USA). Cloning was confirmed by PCR (Fig 1) and sequencing, primer details are available in Table 1. Recombinant plasmids containing the synthetic genes of TES-30 and TES-120 were used to transform *Escherichia coli* BL21 Star (Invitrogen, USA) that was then cultivated in Luria Bertani (LB) medium (Tryptone 10 g/L, Yeast Extract 5 g/L, 170 mM, Synth, Brazil) supplemented with 100 μg/mL of ampicillin (Sigma-Aldrich, USA) at 37° C in a rotatory shaker at 180 rpm. When the culture reached an optical density of 0.6 at 600 nm measured by spectrophotometer (Hitachi U-1800, Japan), protein expression was induced for 3 hours at 37° C with isopropyl β-D-1-thiogalactopyranoside (Ludwig, Brazil) at the final concentration of 0.5 mM. Chemical lysis with buffer (50 mM Na2HPO4, 0.5 M NaCl, pH 8.0, Synth, Brazil) containing lysozyme (Sigma-Aldrich) and sonication (Cole Parmer, Ultrasonic Homogenizer 4710) were used to release the protein to the medium. After centrifugation (10.000 *g*/10minutes), the pellet was washed with PBS-T (10 mM sodium phosphate, 0.15 M NaCl, 0.05% Tween 20, pH 7.5, Synth, Brazil), and solubilized with a 6 M urea phosphate buffer (6 M Urea, 50 mM Na2HPO4, 0.5 M NaCl, 5 mM imidazole, pH 8.0, Synth, Brazil). Proteins were purified with ÄKTA Start and HisTrap HP purification columns (GE Healthcare Life Sciences, USA), using 6 M urea phosphate buffer and 6 M urea elution phosphate buffer (6 M Urea, 50 mM Na2HPO4, 0.5 M NaCl, 200 mM imidazol, pH 8.0, Synth, Brazil). The sizes of the expressed target proteins were determined by sodium dodecyl sulfate-polyacrylamide gel electrophoresis (SDS-PAGE) analysis. The proteins were further quantified by Pierce BCA kit (Thermo Fisher Scientific, USA).

**Table 1:** Primer details of rTES-30 and rTES-120 for confirmation of cloning in expression vector pAE.

Protein	Forward Primer Sequence	Reverse Primer Sequence	Size of Product (pairs of base)
rTES-30	5’- CCCAAGCTTTCACAAAGGTCTCTTACA–3’	5’-CCCAAGCTTTCACAAAGGTCTCTTACA–3’	641
rTES-120	5’-AGCGGATCCATGTCTTCTTCTTCTTC–3’	5’-TTCAAGCTTCTAGCAGAATCCACAGGTCAA–3’	502

### Sample collection

Samples were collected from three distinct serum panels. Serum panel 1 samples were collected from 48 children between 2–8 years old at the Hospital Universitário by Dr. Miguel Riet Corrêa Jr in Rio Grande—RS, Brazil. Within this collection, 22 children were seropositive for *Toxocara* spp. infection and 26 children were seronegative. Positives and negatives samples were diagnosed by clinic data, seropositivity to *Toxocara* spp. (via native TES ELISA and Western blotting confirmation) and three epidemiological factors: eosinophilia, contact with young dogs/cats, and geophagy. Serum panel 2 were collected samples from 42 adults (age > 21 years) at the Parasitology Laboratory of the Universidade Federal do Rio Grande and Universidade Federal de Pelotas. 23 adults were seropositive for *Toxocara* spp. infection and 19 seronegatives were adults. Positives and negatives samples were diagnosed by clinic data, seropositivity to *Toxocara* spp. (via native TES ELISA and Western blotting confirmation) and two epidemiological factors: eosinophilia and contact with young dogs/cats. Serum panel 3 samples were collected from Laboratório de Soroepidemiologia e Imunobiologia do Instituto de Medicina Tropical de São Paulo (IMT-SP) of Universidade de São Paulo. All sera were found to be seronegative for *Toxocara* spp. infection based on epidemiology factors and laboratory assays. Other parasites were diagnosed from clinic data, epidemiology, and laboratory assays, revealing 40 cases with parasite infections (9 patients with *Ascaris lumbricoides*, 2 patients with *Trichuris trichiura*, 4 patients with *Ancylostoma duodenale*, 16 patients with *Strongyloides stercoralis*, 6 patients with *Hymenolepis nana*, and 3 patients with *Fasciola hepatica*) and 40 cases without.

### Indirect ELISA

To verify the antigenicity of the recombinant proteins, we used ELISA. ELISA protocol was optimized prior to the study. Each well of the 96-well flat-bottomed microtiter plate (Nunc Immuno Maxisorp, Thermo Fischer Scientific, USA) was coated with 100 μL of each recombinant antigen at the optimum concentration (50 ng) for each antigen in 0.02 M bicarbonate buffer, pH 9.6. The plates were then covered and incubated at 4°C overnight. The plates were washed with PBS-T, to remove unadsorbed antigen. After a washing step of three washes for 5 min each with PBS-T, each well was blocked with 5% dry-milk (Nestle, Sweden) PBS-T solution for 1 h at 37°C. The plates were again washed as previously described, followed by the addition of sera samples (100 μL, 1:150 in PBS-T, duplicate wells), and incubated at 37°C for 1 h. After the washing step, monoclonal anti-human IgG-horseradish peroxidase (Thermo Fisher Scientific, USA) were added at an optimized dilution (1:5000) in PBS-T, or mouse anti-human IgG2 (Sigma Aldrich, USA) were added at an optimized dilution (1:2500) in PBS-T, or mouse anti-human IgG4 (Sigma Aldrich, USA)) were added at an optimized dilution (1:2500) in PBS-T, followed (in case of IgG2 and IgG4) by anti-mouse IgG-horseradish peroxidase (Thermo Fisher Scientific, USA) and incubated at 37°C for 1 hour. Following a final washing step, o-phenylenediamine dihydrochloride substrate (Sigma Aldrich, USA) was added and the ODs were measured after 15 min as absorbance at 450 nm using an ELISA spectrophotometer (Biocrom EZ Read 400, United Kingdom). The OD readings were blanked with the PBS-T, and the cutoff value was used to discriminate between the positive and negative results. This cutoff value was based on the results of a ROC statistical analysis: 0.3415 for rTES-30/IgG; 0.23 for rTES-120/IgG; 0.083 for rTES-30/IgG2; 0.093 for rTES-120/IgG2; 0.076 for rTES-30/IgG4; and 0.0965 for rTES-120/IgG4.

### Western blotting assay

Lastly, a Western Blotting assay was used to confirm ELISA close to cut-off absorbance (borderline). The rTES-30 (20 μg/ml) was applied in a 12% SDS-PAGE and electrotransferred onto a nitrocellulose membrane (GE Healthcare Life Sciences, USA) by using a transblot apparatus (Bio-Rad, USA) at 4°C overnight. Protein transfer to membrane was checked with Ponceau S staining (Sigma-Aldrich, USA). The membrane was cut into strips and blocked with 5% dry-milk (Nestle, Sweden) PBS-T solution for 1 h. The strips were then incubated with serum/sera samples (diluted 1:200 in PBS-T) at 4°C overnight, followed by monoclonal anti-human IgG-horseradish peroxidase (Thermo Fisher Scientific, USA) were added at an optimized dilution (1:5000) in PBS-T and incubated at 37°C for 1 hour. Between each step, the strips were washed with PBS-T during 5 minutes. Finally, DAB Solution (0.025% 3,3′-Diaminobenzidine, 0.0009% H_2_O_2_ and 0.05M Tris/HCl-solution, Sigma Aldrich, USA) were used to develop the blots. Statistical analysis was performed by Pearson chi-square, two-way ANOVA and ROC curve in GraphPad software.

### Ethics

This study was approved by the Research Ethics Committee in the Health Area, FURG (protocol no. 102/2012 and 100/2010).

## Results and discussion

The expression of our recombinant proteins yielded an 18 kDa protein (rTES-30) and a 24 kDa protein (rTES-120) obtained in inclusion bodies at the concentrations of 17.04 mg/L and 28.56 mg/L, respectively. This protein was stored in a ready-to-use state in urea buffer, similar to Farmer et al. (2017) study [18].

The protein bands were confirmed in a Western Blotting assay. We observed a higher purity of recombinant protein than was published by Mohamad et al. (2009) and Anderson at al. (2015). We achieved this purity by inserting a double histidine tag at each extremity of the protein (5’ and 3’), increasing its affinity to the chromatography column [8,19].

The overall sensitivity and specificity of recombinant proteins is shown in Table 2 and Table 3. There was no benefit associated with using both proteins at the same time, as only one sample reacted with rTES-120 and not with rTES-30 (*p* = 0.8152).

**Table 2:** Optimum sensibility and specificity for each protein and antibody after receiver operating characteristic (ROC) analysis.

Protein/Antibody	Positive Samples (Sensitivity)	Negative Samples (Specificity)
rTES-30/IgG	38/45 (84.4%)	45/45 (100%)
rTES-30/IgG2	4/45 (8.9%)	40/45 (88.9%)
rTES-30/IgG4	10/45 (22.2%)	44/45 (97.8%)
rTES-120/IgG	34/45 (75.6%)	45/45 (100%)
rTES-120/IgG2	6/45 (13.3%)	43/45 (95.6%)
rTES-120/IgG4	23/45 (51.1%)	44/45 (97.8%)

**Table 3:** Specificity of recombinant proteins against a variety of common geohelminths.

	Cross-Reactivity^a^					
	*Ascaris lumbricoides*	*Trichuris trichiura*	Ancylostomids	*Strongyloides stercoralis*	*Hymenolopis nana*	*Fasciola hepatica*
rTES-30	0/9 0%	0/2 0%	0/4 0%	1/16 6.25%	1/6 16.67%	0/3 0%
rTES-120	2/9 22.22%	0/2 0%	0/4 0%	0/16 0%	1/6 16.67%	0/3 0%

a) Cut-off for each antigen was calculated by ROC curve assay of 40 *Toxocara* spp. negative patients.

After categorizing the samples by age, we observed a major change in sensitivity in some protein/antibody combinations, as shown in Table 4.

**Table 4:** Sensitivity and specificity for each protein and antibody after ROC analysis, separated by adult and child samples.

Protein/Antibody	Adult Positive Samples (Sensitivity)	Adult Negative Samples (Specificity)	Children Positive Samples (Sensitivity)	Children Negative Samples (Specificity)
rTES-30/IgG	20/23 (87%)	19/19 (100%)	18/22 (81.8%)	26/26 (100%)
rTES-30/IgG2	1/23 (4.3%)	18/19 (94.7%)	1/22 (4.5%)	25/26 (96.2%)
rTES-30/IgG4	6/23 (26.1%)	19/19 (100%)	1/22 (4.5%)	26/26 (100%)
rTES-120/IgG	22/23 (95.7%)	19/19 (100%)	14/22 (63.6%)	26/26 (100%)
rTES-120/IgG2	2/23 (8.7%)	18/19 (94.7%)	4/22 (18.2%)	25/26 (96.2%)
rTES-120/IgG4	16/23 (69.6%)	19/19 (100%)	6/22 (27.3%)	26/26 (100%)

In this study, we analyzed the diagnostic potential of *T*. *canis* recombinant proteins in more detail, addressing questions raised by previously studies regarding the use of antibody classes for TES antigens and the effects of patient age on sensitivity and specificity.

Overall, we disagree with Mohamad et al. (2009) and Watthanakulpanich et al. (2008), both of which find a higher sensitivity using a combination of proteins to serodiagnose *Toxocara* spp. infections [8,19], while our study favors the use of only one protein (rTES-30). We found that sensitivity and specificity did not improve with the combination of both proteins (*p* = 0.8152), so we recommend the single protein to reduce the associated production cost.

Another difference is that we found a lower sensitivity associated with IgG4 and IgG2, while previous studies found these to be the most sensitive and specific antibodies for toxocariasis. This discrepancy could be attributable to differences in population; this study was performed in Brazil with a climate, culture, and genetic features of *Toxocara* spp. that likely are different in Malaysia, Thailand, and Scotland [8,19,20]. Moreover, we opted to use total IgG instead of subgroups to reduce the cost of diagnosis, as total IgG is less expensive than a specific IgG subgroup (from any given manufacturer).

We also observed a major increase in IgG4 sensitivity in adults over children for both rTES-30 (4.5% in children to 26.1% in adults, *p* = 0.0959) and rTES-120 (27.3% in children to 69.6% in adults, *p* = 0.0072). This variable could explain why Mohamad et al. (2009) found that IgG4 was the most sensitive: that study used an adult population [8]. Among all immunoglobulins, IgG4 is produced the least and is the last to be produced, so it is positive in chronic diseases only [21]. As children do not yet have a mature immune system and are more likely to have contracted the infection recently, the immune system has less time to produce a viable IgG4 mediated-response [14,21].

In addition, Watthanakulpanich et al. (2008) used native TES to infer that IgG2 was the most sensitive and specific. Native TES is highly glycosylated, unlike in our expression system (*Escherichia coli*), so it cannot be expected to induce the same reactivity. Also, Watthanakulpanich et al. (2008) did not preabsorb serum with *Ascaris* sp. so cross-reactivity with other geohelminths could have influenced the results [20,22].

Regardless, our main finding is the difference in sensitivity between adult and pediatric age groups. When we divided our sample pool between children (age 2–8 years) and adults (age > 21 years), we discovered that rTES-120 sensitivity correlated negatively with age, decreasing from 95.7% in adults to 63.6% in children (*p* = 0.0098). Meanwhile, rTES-30 sensitivity was not affected by age: 81.8% in children and 87% in adults (*p* = 0.6995).

Native TES-120 is a mucin, a heavily glycosylated protein, so its lower sensitivity in children could mimic the situation that occurs in encapsulated bacteria vaccinations, such as those for *Haemophilus influenzae*, *Streptococcus pneumoniae*, and *Neisseria meningitidis*. The polysaccharides of these bacteria in isolation do not illicit a proper immune response in young children because they lack a mature T-independent response. Mucin has a similarly high concentration of carbohydrates as polysaccharides, so this could explain the discrepancy we found in specificity between children and adults [23–25]. Alternatively, native TES-30 is a C-type lectin, with serine proteinase activity and a lower ratio of carbohydrates to protein than native TE-120. It is associated with the larval migratory process and infection; hence, it is one of the first proteins to be produced and recognized by the pediatric immune system, explaining its higher sensitivity [22,26].

**Fig 2:**
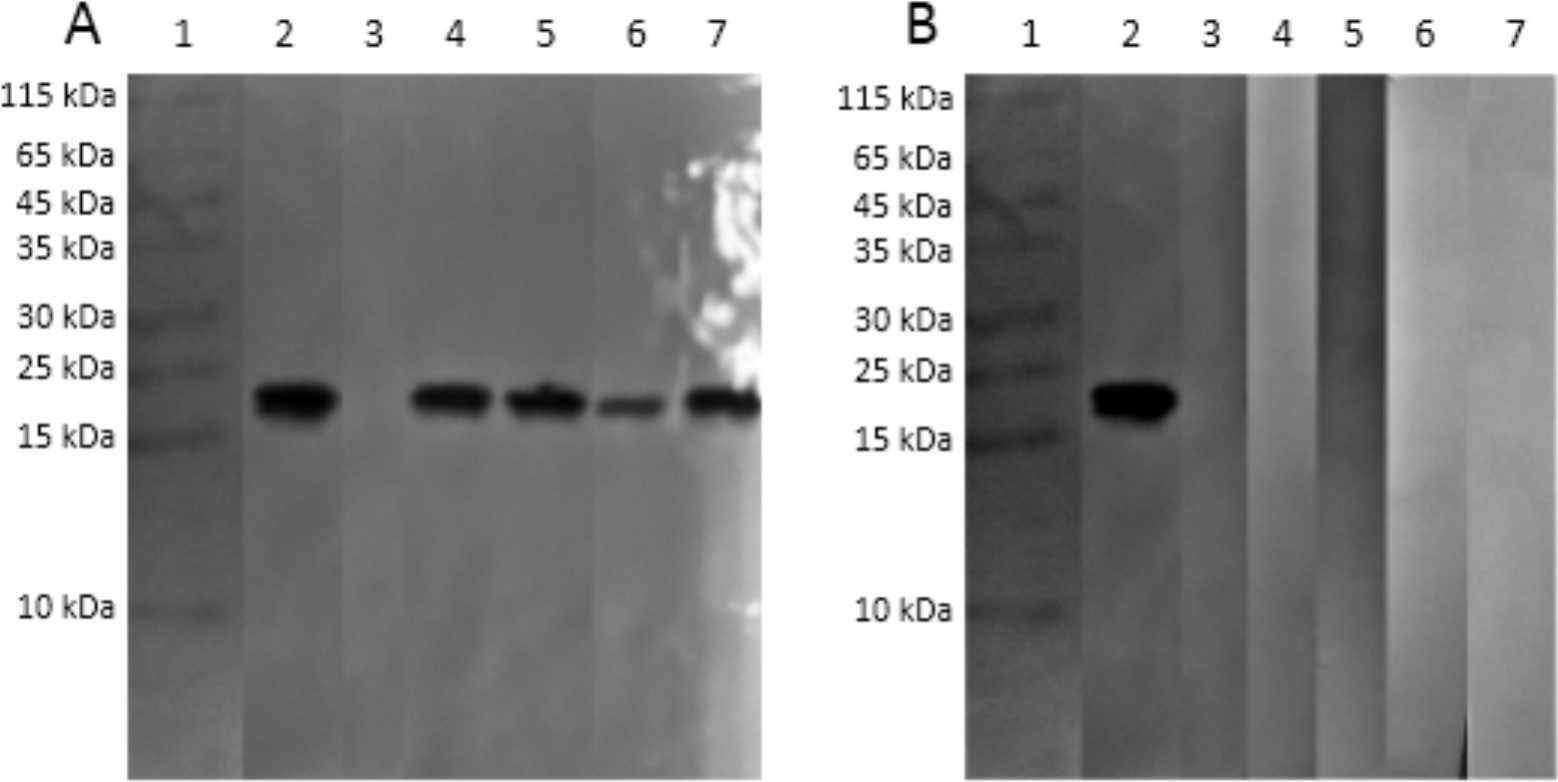
**(A) Western blot analysis of rTES-30 antigen probed with serum that was close to the ELISA cut-off.** Lane 1: PageRuler Prestained Protein Ladder (Thermo Fischer Scientific, USA). Lane 2: positive/loading control. Lane 3: negative control. Lanes 4–7: sera from four different Toxocara-seropositive patients. **(B) Western blot analysis of rTES-30 antigen probed with serum that was close to the ELISA cut-off.** Lane 1: PageRuler Prestained Protein Ladder (Thermo Fischer Scientific, USA). Lane 2: positive/loading control. Lane 3: negative control. Lanes 4–7: sera from apparently healthy people.

In this study, we defend the use of rTES-30 as a sole recombinant protein for the serodiagnosis of human *Toxocara* spp. We achieved an 84.4% (38/45) sensitivity with ELISA regardless of the patient’s age and reached 97.8% (44/45) with Western Blotting confirmation (Fig 2). The discrepancy between ELISA and Western Blot results was expected as ELISA determines that a sample is positive or negative based on an arbitrary number (cut-off), while a Western Blotting assay relies on a simple observation of an adequate size band [27–29]. We suggest that ELISA is still the optimal first-line technique because it is relatively inexpensive, making it a good fit for a neglected disease [30], but a Western Blotting assay should be utilized as a confirmation technique in sera with absorbances that are close to the cut-off, this has also been suggested by Maraghi et al. (2012) [31]. We also recommend a Western Blotting assay to rule out other causes (infectious or non-infectious) for a specific symptom or finding, such as hypodense or hyperintense brain lesions (neurotoxocariasis), and to evaluate immunocompromised patients (e.g., to rule out toxoplasmosis, schistosomiasis, and others common opportunistic diseases) [2,32–35].

Moreover, even with an ELISA sensitivity of 95.7% in adults, no statistical difference was seen between rTES-120 and rTES-30 (p = 0.6078). Our finding is confirmed by a recent study from the Centers for Disease Control and Prevention (CDC). A U.S. national survey performed with recombinant rTc-CTL-1 (99.9% identity to rTES-30) found a 5.1% prevalence in one developed country [18]. That study used a Luminex bead assay, similar to but more expensive than an indirect ELISA as the protein rTc-CTL-1 needed to be pre-coated with color-coded beads.

Furthermore, we expected high specificity (>94%) of every recombinant protein as has been documented previously [8]. It is important to state that we expect that the recombinant proteins have cross-reactivity with *T*. *cati* TES [9], homolog to *T*. *canis*, this cross-reactivity is not a specificity issue and it is important that both recombinant proteins detect *T*. *cati* sorology, as the infection remains the same with potential to cause visceral and ocular toxocariasis [36]. While Mohamad et al. (2009) studied the specificity of recombinant proteins against *Ascaris lumbricoides*, *Trichuris trichiura*, lymphatic filariasis, amoebiasis, and toxoplasmosis, we studied the specificity of recombinant proteins against strongyloidiasis, hymenolepiasis, and fasciolosis—three diseases that affect patients of developing countries and could potentially jeopardize the diagnosis of toxocariasis with native TES [8,37,38]. Strongyloidiasis is particularly noteworthy as *Strongyloides stercoralis* and *Toxocara* sp. are endemic parasites in tropical countries (e.g., Brazil) and are often co-morbid in patients with human immunodeficiency virus and neuroparasites. In these situations, it is therefore ideal to have a diagnostic assay without cross-reactivity issues [31,39–41].

## Conclusions

In conclusion, our study favors the use of rTES-30 with total IgG as the primary antibody in an indirect ELISA assay as a tool for epidemiological human studies.
